# Supplementary material for: A Charcot-Marie-Tooth-Causing Mutation in HSPB1 Decreases Cell Adaptation to Repeated Stress by Disrupting Autophagic Clearance of Misfolded Proteins
Source: Cells. 2022 Sep 15;11(18):2886. doi: 10.3390/cells11182886 (PMC9496658; doi:10.3390/cells11182886)
Supplement: Supplementary file 1 [file cells-11-02886-s001.zip › cells-1849914-supplementary.pdf]

A Charcot-Marie-Tooth-causing Mutation in HSPB1 Decreases Cell Adaptation to Repeated Stress by Disrupting Autophagic Clearance of Misfolded Proteins

Xuelian Zhang[1], Yaru Qiao[1], Ronglin Han[1], Yingjie Gao[2], Xun Yang[1], Ying Zhang[1], Ying Wan[1], Wei Yu[3]*, Xianchao Pan[2]* and Juan Xing[1]*

[1] Department of Pathophysiology, School of Basic Medical Science, Southwest Medical University, Luzhou, 646000, China;
[2] Department of Medicine Chemistry, School of Pharmacy, Southwest Medical University, Luzhou, 646000, China;
[3] State Key Laboratory of Genetic Engineering, School of Life Sciences, Zhongshan Hospital, Fudan University, Shanghai, 200438, China.

* Correspondence: xingjuan217@swmu.edu.cn (J.X.); panxc@swmu.edu.cn (X.P.); yuw@fudan.edu.cn (W.Y.)

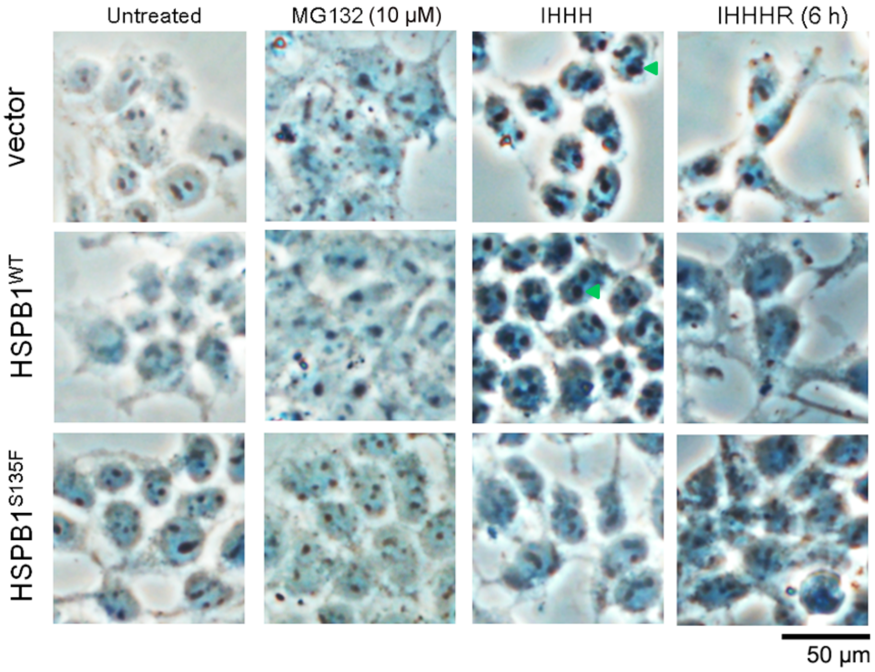

Figure S1. Stress-induced aggresomes in HEK293T cells were shown as dark inclusions by phase contrast microscope (indicated by green triangles). Dark inclusions in heated cells with vector or HSPB1^{WT} disappeared after recovery of 6 h. There are some dark dots scattered in HSPB1^{S135F} cells, but obviously stress-induced formation of aggresomes was absent. Scale bar: 50 μm.

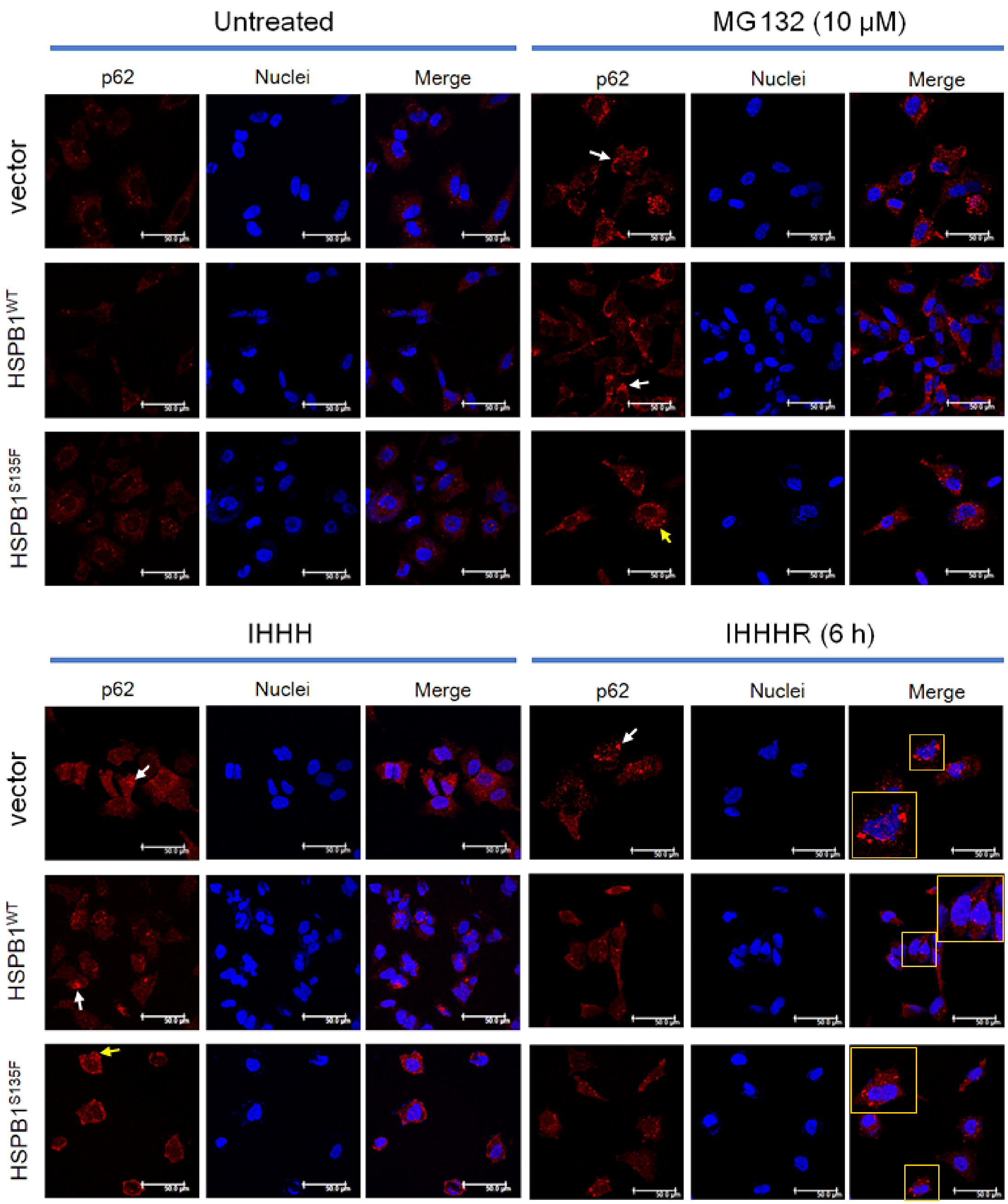

Figure S2. HSPB1^{S135F} prevents perinuclear transport of p62 in SH-SY5Y cells under stress condition. Stress induced perinuclear accumulation of p62 punctuate inclusions in vector and HSPB1^{WT} cells was indicated by white arrows. Yellow arrows show uniform distribution of p62 in HSPB1^{S135F} cells. After recovery of 6 h, fewer punctuate inclusions was observed in HSPB1^{WT} cells compared with vector and HSPB1^{S135F} cells. Besides, p62 punctuate inclusions located around nuclear in cells with vector, while away from nuclear in cells with HSPB1^{S135F}. Insets are magnified images. Scale bars: 50 μm.
